# Silent but Deadly: A Case of Obtuse Marginal Artery Occlusion

**DOI:** 10.7759/cureus.85561

**Published:** 2025-06-08

**Authors:** Austin M Miller, Vishal Tharani, Erin Fraser, Howard W Levitin

**Affiliations:** 1 Emergency Medicine, OhioHealth Doctors Hospital, Columbus, USA

**Keywords:** atypical chest pain, cardiac arrest, first obtuse marginal branch of left circumflex artery, non-st segment elevation myocardial infarction (nstemi), omi, st-segment elevation myocardial infarction (stemi)

## Abstract

The obtuse marginal artery (OMA) is a key branch of the left circumflex coronary artery, supplying blood to the lateral wall of the left ventricle. OMA occlusions can result in myocardial ischemia and serious cardiac events, often presenting with subtle or atypical electrocardiogram (EKG) changes, unlike the more pronounced alterations observed with left anterior descending artery occlusions. This case report discusses a 57-year-old female presenting with chest pain and shortness of breath, indicative of acute coronary syndrome. The patient's initial EKG showed occasional premature ventricular contractions (PVCs), T-wave flattening in aVL, and 1 mm ST elevation in V6, but otherwise, it was normal appearing. Despite not having typical EKG abnormalities, the patient suffered a cardiac arrest. Coronary angiography confirmed 100% stenosis of the first OMA branch, which was successfully treated with aspiration thrombectomy and stent placement. The patient did not have collateral coronary vessels, and therefore, did not have a role in making the patient's EKGs difficult to interpret. This case emphasizes the challenges posed by OMA occlusions, underscoring the necessity for a high index of suspicion during the early stages of a patient’s presentation for prompt diagnosis and treatment.

## Introduction

The obtuse marginal artery (OMA) is a crucial branch of the left circumflex coronary artery, supplying blood to the lateral wall of the left ventricle. Blockages in the OMA can lead to myocardial ischemia and serious cardiac events. Patients with OMA blockages may experience chest pain, shortness of breath, or other ischemic symptoms, which can progress to acute coronary syndrome if left untreated. These symptoms are nonspecific and may overlap with other coronary syndromes.

Detecting occlusions in the OMA on an EKG can be challenging due to the specific regions supplied by this artery. The lateral wall of the left ventricle is affected, often resulting in subtle EKG changes. Occasionally, due to anatomical variations, the OMA may also supply the anterior and inferior walls, though this is rare [1]. Unlike occlusions in the left anterior descending artery, which typically show clear EKG changes, such as ST-segment elevations in the anterior leads, OMA occlusions may result in less pronounced or even undetectable changes [2-4]. Subtle ST-segment changes in the lateral leads can be overlooked, particularly in cases of partial occlusions or when compensatory collateral circulation is present. A study examining EKG findings in various coronary occlusive events found that ST elevation in lead aVL, along with ST depression in lead V2, can predict obstruction of the first obtuse marginal branch [5]. Previous ischemic changes can further complicate EKG interpretation and diagnosis.

OMA occlusions are typically confirmed through coronary angiography. Early detection and management are critical to prevent complications and enhance cardiac outcomes. This case discusses a patient diagnosed with an OMA branch occlusion via coronary angiography after experiencing cardiac arrest in the emergency department.

## Case presentation

A 57-year-old female with a medical history of depression, vertigo, and a thyroid disorder presented to the emergency department (ED) with chest pain radiating to her left arm. The pain started approximately 30 minutes prior to her arrival. According to the patient and her daughter, who were in separate vehicles, the patient had to pull over due to chest pain and palpitations, which led her daughter to call emergency medical services (EMS). EMS administered 324 milligrams (mg) of aspirin and one nitroglycerin tablet, providing some relief.

The patient reported that her chest pain had decreased since it began. She also experienced tingling in her left arm and shortness of breath, which she attributed to anxiety. She denied having symptoms such as nausea, vomiting, headache, dizziness, blurry vision, abdominal pain, recent illness, dysuria, hematuria, lower extremity edema, or back pain. The patient is a former smoker who quit in 2007 and denied using drugs, alcohol, or vaping. Her current medications included diazepam, fluticasone propionate nasal spray, and naproxen, and she had no known drug allergies.

Her initial vital signs included a blood pressure of 112/68 millimeters of mercury (mmHg), a temperature of 97.7 degrees Fahrenheit, a pulse of 65 beats per minute, a respiratory rate of 16 breaths per minute, and an oxygen saturation of 95% on room air. The physical examination revealed tachypnea, which was contradictory to the initial respiratory rate documented, and an anxious mood, but was otherwise unremarkable. Notable findings included the absence of lower extremity edema, a regular heart rate, normal breath sounds, and no signs of infection, cyanosis, neurological symptoms, or abdominal pain.

**Figure 1:**
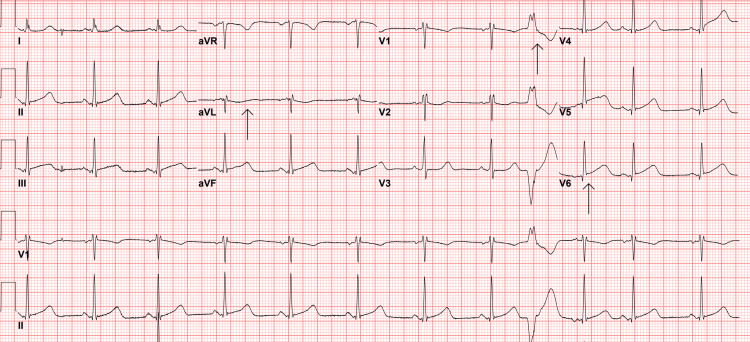
Patient’s first EKG on arrival at the emergency department

Given her symptoms, an EKG was performed upon arrival (Figure 1), and laboratory tests were ordered (Table 1). The initial EKG revealed sinus rhythm, occasional premature ventricular contractions (PVCs), T-wave flattening in aVL, and 1 mm ST elevation in V6. Based on her clinical presentation, symptoms, and medical history, the differential diagnoses included acute coronary syndrome (ACS), pulmonary embolism, pneumonia, spontaneous pneumothorax, abdominal aortic aneurysm, pancreatitis, myocarditis, pericarditis, and non-cardiac chest pain.

**Table 1:** Initial laboratory results

Lab	Value (range)
White blood cell	6.48 K/mcL (4.5 - 11 K/mcL)
Red blood cell	3.9 M/mcL (4.0 - 5.2 M/mcL)
Hemoglobin	12 g/dL (12.0 - 16.0 g/dL)
Hematocrit	35.5% (36.0 - 46.0%)
Mean corpuscular volume	91 fL (80.0 - 100.0 fL)
Platelet	230 K/mcL (150 - 400 L/mcL)
Sodium	142 mmol/L (135 - 145 mmol/L)
Potassium	4 mmol/L (3.5 - 5.1 mmol/L)
Chloride	105 mmol/L (98 - 108 mmol/L)
Bicarbonate	24 mmol/L (21 - 32 mmol/L)
Anion Gap	17 mmol/L (10 - 20 mmol/L)
Glucose	108 mg/dL (65 - 99 mg/dL)
Blood Urea Nitrogen	15 mg/dL (8 - 25 mg/dL)
Creatinine	0.79 mg/dL (0.40 - 1.10 mg/dL)
Total Protein	7.1 g/dL (6 - 8 g/dL)
Albumin	4.3 g/dL (3.2 - 5.2 g/dL)
Troponin	9 ng/L (<= 14 ng/L)

Approximately 30 minutes after the initial evaluation, a Code Blue was called for the patient's room when her daughter exited, shouting for help. Staff members observed the patient experiencing a seizure-like event and found no detectable pulse. This seizure-like event was likely a myoclonic response due to the patient being in ventricular fibrillation (V-fib). Initial telemetry revealed V-fib, which quickly deteriorated into asystole. The nursing staff immediately initiated cardiopulmonary resuscitation (CPR), brought a crash cart to the bedside, and applied defibrillation pads to the patient. One milligram of epinephrine was administered during the cardiac arrest.

**Figure 2:**
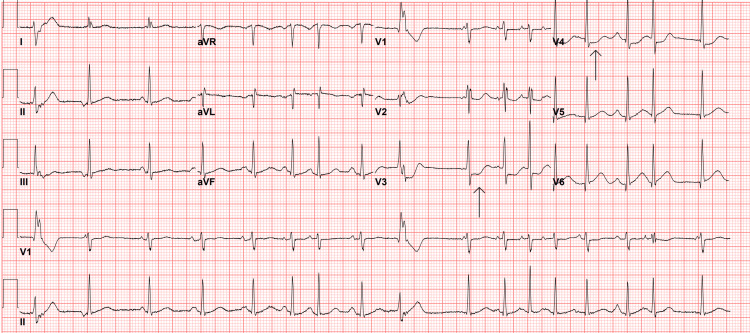
Post-cardiac arrest EKG

After two minutes of CPR, telemetry indicated V-fib and the patient had no palpable pulse. The patient was defibrillated once, and a subsequent pulse check confirmed the return of spontaneous circulation (ROSC). A repeat EKG revealed sinus tachycardia, frequent PVCs, occasional premature atrial contractions (PACs), and new ST depressions in leads V3 and V4 (Figure 2). Although this EKG did not meet ST-segment elevation myocardial infarction (STEMI) criteria, interventional cardiology was consulted.

Given the patient's V-fib arrest, ongoing chest pain, and new ST abnormalities on the post-cardiac arrest EKG, the decision was made to activate the catheterization lab. The patient received a dose of 5000 units of heparin and was started on an amiodarone infusion following an initial bolus of 150 mg. Amiodarone was used, as it is the drug we are most familiar with using in post-cardiac arrest patients in our facility.

**Figure 3:**
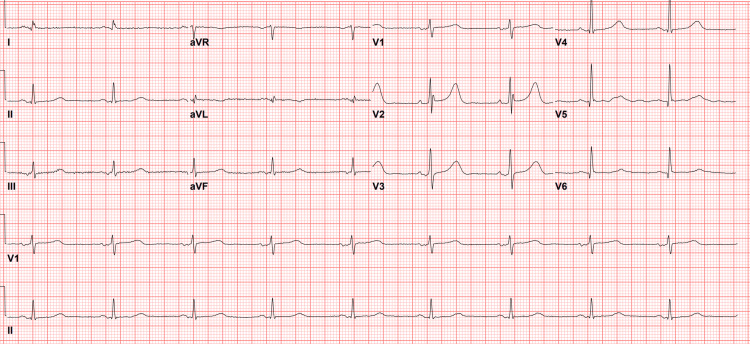
EKG immediately before catheterization

Before catheterization, a 1-liter fluid bolus of 0.9% sodium chloride was administered intravenously to address the patient's suboptimal blood pressure of 94/58 mmHg. A third EKG revealed no acute ST elevations or depressions but some nonspecific T-wave abnormalities (Figure 3). The QTc on this EKG was slightly prolonged at 491 milliseconds, prompting the administration of 2 grams of magnesium to help shorten that interval and prevent dysrhythmia.

**Figure 4:**
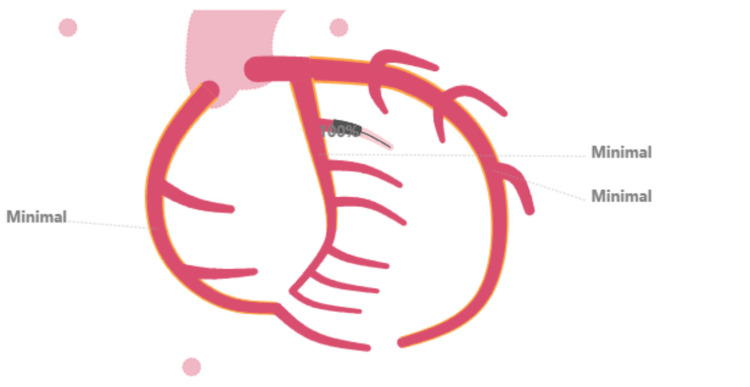
Illustration of coronary arteries and site of occlusion

The patient successfully underwent cardiac catheterization, which involved aspiration thrombectomy and the placement of a drug-eluting stent guided by intravascular ultrasound. During the procedure, a proximal edge dissection of the artery occurred, which was treated with a second drug-eluting stent. An acute thrombotic occlusion of the first obtuse marginal artery branch was identified as the culprit lesion, demonstrating 100% stenosis (Figure 4). Following the procedure, the patient was admitted to the intensive care unit (ICU) and was discharged home two days later in stable condition.

## Discussion

In this case, a 57-year-old female presented with symptoms suggestive of ACS. The initial EKG did not reveal any acute ST/T wave abnormalities typical of a STEMI, underscoring the challenges of detecting OMA occlusions through standard EKGs due to their subtle presentations (2-4). This is supported by studies such as that of Birnbaum et al., which found that ST elevation in lead aVL coupled with ST depression in V2 could indicate OMA branch obstruction [3]. ST elevation, specifically in lead aVL, can be indicative of the first obtuse marginal or lateral circumflex infarction. These changes are often subtle and easily overlooked [5]. These alterations were not evident in the patient’s initial EKG.

Other case reports, including one by Aoyama et al., documented similar challenges in diagnosing OMA occlusions. They described a non-ST-segment elevation myocardial infarction (NSTEMI) identified using spectral detector-based computed tomography (CT) when the EKG did not indicate ischemia [6]. Initially, the patient exhibited a right bundle branch block but no ST elevation. The diagnosis was confirmed after the patient experienced sudden chest pain and dyspnea, accompanied by elevated troponin levels and a perfusion defect in the posterolateral left ventricular myocardium observed on CT images.

Additionally, a case by Livesay and Johnson highlighted the complexity of diagnosing myocardial infarctions in patients with atypical presentations. They reported a patient with Moyamoya disease who presented with a STEMI due to a 90% stenosis of the first obtuse marginal branch and new 40-50% restenosis of a previous distal right coronary artery (RCA) stent [7]. This underscores the variability in EKG presentations depending on individual anatomical and pathological differences.

It is also important to note the low sensitivity of STEMI. The sensitivity of STEMI is 43.6%, based on three studies verifying the diagnostic accuracy of the current STEMI criteria [8]. A false-negative STEMI (non-STEMI/NSTEMI) does not rule out occlusive myocardial infarction (OMI). This underscores the need for a shift toward diagnosing myocardial infarctions as either OMI or non-occlusive myocardial infarction (NOMI) [9].

In this case, the patient experienced a cardiac arrest in the emergency department, likely secondary to an acute OMA occlusion. The OMA supplies the lateral left ventricular wall, which is likely why the patient developed V-fib. Despite initial and subsequent EKGs still not meeting STEMI criteria, the patient was determined to have had a high-risk NSTEMI, making activating the catheterization lab a crucial therapeutic step. Notably, 25% of NSTEMI patients with OMI have a fully occluded artery, and there is nearly double the mortality rate in these patients compared to those with NSTEMI and no OMI [10]. This can make risk stratification more difficult, especially in the ED environment. This may also mean that more patients with NSTEMI need emergent coronary angiography rather than simply treating with anti-thrombotic medications. Coronary angiography confirmed a 100% stenosis of the patient's first obtuse marginal artery branch, which was successfully treated with aspiration thrombectomy and drug-eluting stent placement.

## Conclusions

This case, along with the referenced studies, highlights the importance of maintaining a high index of suspicion for OMA occlusions and other EKG “silent” occlusions, particularly in patients with lateral wall ischemic symptoms and atypical or normal-appearing EKG presentations. EKG "silent" refers to occlusions without specific ST-segment changes or meeting typical STEMI criteria. Early detection and intervention are critical to preventing severe complications, such as myocardial infarction, and improving patient outcomes, including reducing mortality and preserving cardiac function. These cases collectively emphasize the need for comprehensive diagnostic approaches, including advanced imaging techniques (spectral CT or cardiac MRI) and coronary angiography, to accurately diagnose and manage coronary artery occlusions that might otherwise be missed. Cardiology should also be involved early in these cases since other atypical presentations that lead to myocardial infarction must be considered.
